# Antithrombotic Management for Atrial Fibrillation Patients Undergoing Percutaneous Coronary Intervention or With Acute Coronary Syndrome: An Evidence-Based Update

**DOI:** 10.3389/fcvm.2021.660986

**Published:** 2021-06-28

**Authors:** Shujuan Zhao, Xuejiao Hong, Haixia Cai, Mingzhou Liu, Bing Li, Peizhi Ma

**Affiliations:** ^1^Department of Pharmacy, Henan Provincial People's Hospital, People's Hospital of Zhengzhou University, School of Clinical Medicine, Henan University, Zhengzhou, China; ^2^Department of General Practice, Henan Provincial People's Hospital, People's Hospital of Zhengzhou University, School of Clinical Medicine, Henan University, Zhengzhou, China

**Keywords:** atrial fibrillation, acute coronary syndrome, antithrombotic therapy, non-vitamin K antagonist oral anticoagulants, vitamin K antagonist

## Abstract

Combined antithrombotic regimens for atrial fibrillation (AF) patients with coronary artery disease, particularly for those who have acute coronary syndrome (ACS) and/or are undergoing percutaneous coronary intervention (PCI), presents a great challenge in the real-world clinical scenario. Conventionally, a triple antithrombotic therapy (TAT), which consists of combined oral anticoagulant therapy to prevent systemic embolism or stroke along with dual antiplatelet therapy to prevent coronary arterial thrombosis (CAT), is used. However, TAT has been associated with a significantly increased risk of bleeding. With the emergence of non-vitamin K antagonist oral anticoagulants (NOACs), randomized controlled trials have demonstrated a better risk-to-benefit ratio of dual antithrombotic therapy (DAT) in combination of a NOAC and with a P2Y12 inhibitor than vitamin K antagonist-based TAT. The results of these studies have impacted the recommendations of current international guidelines, which favor a DAT with a NOAC and P2Y12 inhibitor (especially clopidogrel) in this clinical setting. Additionally, aspirin can be administered during the periprocedural period, while the treatment duration of TAT should be as short as possible. In this article, we summarize the up-to-date evidence regarding antithrombotic regimens for AF patients with PCI or ACS, with a specific focus on the optimal approach and critical discussions of key scientific data and future developments for antithrombotic management in these patients.

## Introduction

Atrial fibrillation (AF) is a common atrial arrhythmia, which is particularly prevalent in the elderly. Globally, AF has become a major concern of public health. Patients with AF have increased risks of thromboembolic complications, including stroke and other cardiovascular events (1). Accordingly, antithrombotic therapy has become a cornerstone for the management of patients with AF. However, many AF patients have comorbidities of coronary artery disease (CAD), thus antithrombotic regimens for these patients pose a great challenge. Theoretically, optimal antithrombotic regimen should be effective in decreasing the thrombotic events with remarkably increased bleeding risk. Therefore, physicians must carefully focus on choosing a treatment strategy that can balance the risks of ischemic stroke (IS), thromboembolism, coronary ischemic event recurrence, and stent thrombosis (ST) with the risk of antithrombotic-related bleeding, which makes determination of the optimal antithrombotic regimens and durations a great dilemma in real-world clinical practice (2).

## Background

Conventionally, triple antithrombotic therapy (TAT) that involves an oral anticoagulation (OAC) and dual antiplatelet therapy (DAPT) has been applied over the past decade. However, TAT is connected with a significantly increased risk of bleeding events, and the trade-offs concerning the risk-to-benefit ratio are not entirely clear (3). Subsequently, a dual antithrombotic therapy (DAT) consisting of an OAC and a P2Y12 inhibitor has been shown to confer notably lower bleeding risk and therefore has emerged as an appealing alternative to TAT (4). Recently, four randomized controlled trials (RCTs) have been published regarding the safety and efficiency of non-vitamin K antagonist oral anticoagulants (NOACs) vs. vitamin K antagonist (VKA) on the basis of single or dual antiplatelet agents in AF patients undergoing PCI or with ACS (5–8). The results of these RCTs have provided some novel evidence for the optimization of antithrombotic therapy for these patients.

Accordingly, increasing international guidelines, key updates and consensus processes are regularly released to provide novel evidence and recommendations regarding optimized antithrombotic therapy for AF patients undergoing percutaneous coronary intervention (PCI) or with acute coronary syndrome (ACS). The intersection between AF and those undergoing PCI has been discussed in the 2019 Guideline for the Management of AF from the American College of Cardiology (ACC), American Heart Association (AHA), and Heart Rhythm Society (HRS) (9). In 2020, Chinese Society of Cardiology has published expert consensus document the antithrombotic management of patients with atrial fibrillation and coronary artery disease (10). Later, practical recommendations for the treatment for AF-PCI patients were also issued in the 2020 European Society of Cardiology (ESC) Guidelines for the Diagnosis and Management of AF Developed in Collaboration with the European Association of Cardio-Thoracic Surgery (EACTS) (11). In keeping with the increasing development in the field of AF-PCI, the above documents represent the views from American, European and Chinese experts regarding antithrombotic pharmacotherapy and are endorsed by international scientific associations through practical suggestions and guidelines.

To summarize the available evidence, the present article offers an update to the latest references, recommendations, suggestions, evidence levels and future orientations involving antithrombotic treatment for AF patients receiving PCI or who also have ACS. Furthermore, the latest published guidelines and expert consensus that can provide a general framework for recommendation categories and evidence degrees are also presented, focusing on the practical issues that exist in clinical practice from the North American, European and Chinese points of view. Our intention was to improve the accuracy of evaluation of combined antithrombotic treatment strategies in these patients and to provide a more comprehensive perspective on the administration of NOAC-based regimens for clinicians (12).

## NOAC-Based RCTs: Evidence for AF-PCI/ACS

**Figure 1:**
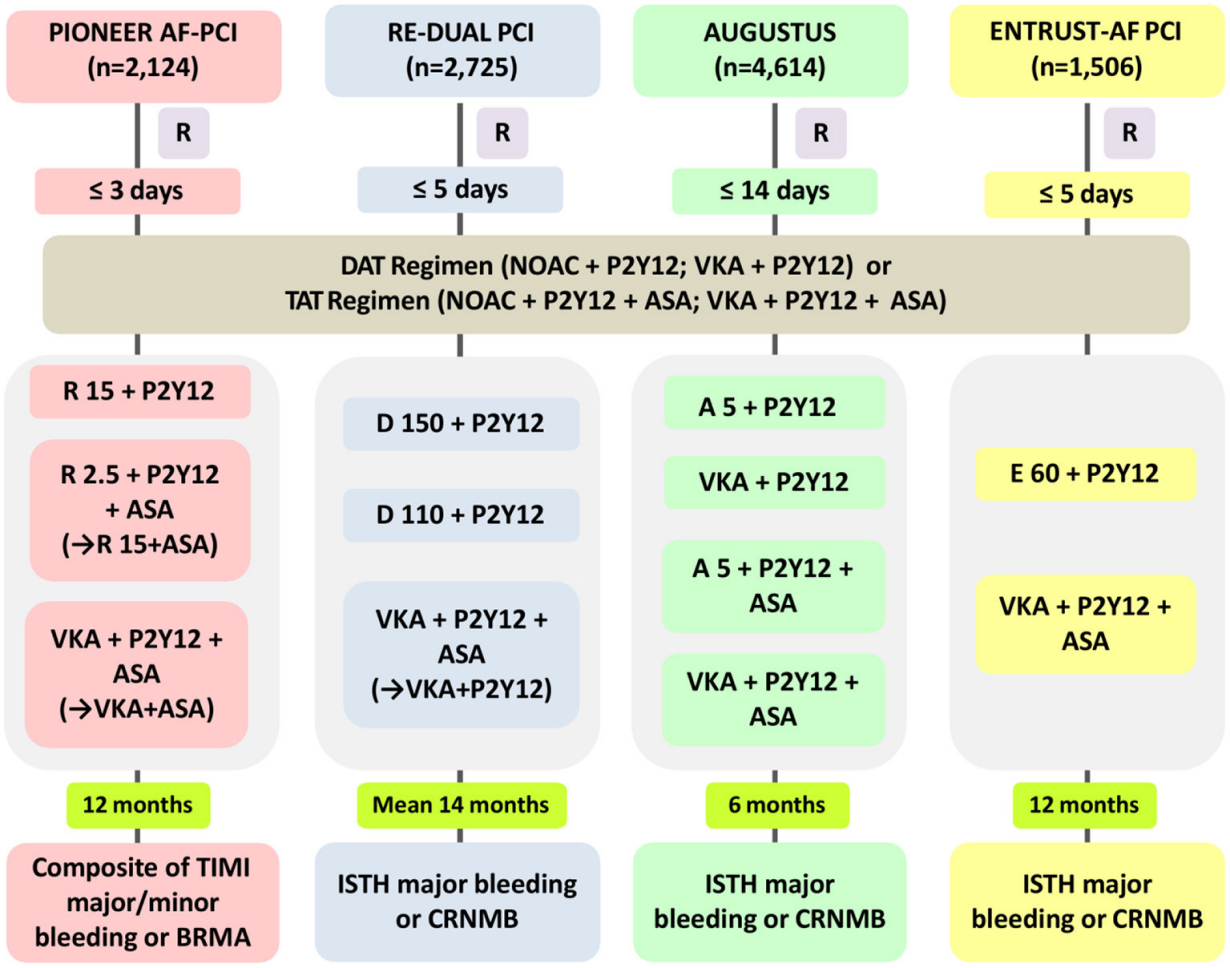
Rationale and design of the four NOAC-based trials in AF patients with ACS or undergoing PCI. NOAC, non-vitamin K antagonist oral anticoagulant; PIONEER AF-PCI, Open-Label, Randomized, Controlled, Multicenter Study Exploring Two Treatment Strategies of Rivaroxaban and a Dose-Adjusted Oral Vitamin K Antagonist Treatment Strategy in Subjects with Atrial Fibrillation who Undergo Percutaneous Coronary Intervention; RE-DUAL PCI, Randomized Evaluation of Dual Antithrombotic Therapy with Dabigatran vs. Triple Therapy with Warfarin in Patients with Non-valvular Atrial Fibrillation Undergoing Percutaneous Coronary Intervention; AUGUSTUS, Open-label, two-by-two Factorial, Randomized Controlled, Clinical Trial to Evaluate the Safety of Apixaban vs. Vitamin K Antagonist and Aspirin vs. Aspirin Placebo in patients with Atrial Fibrillation and Acute Coronary Syndrome and/or Percutaneous Coronary Intervention; ENTRUST-AF PCI, Edoxaban Treatment vs. Vitamin K Antagonist in Patients with Atrial Fibrillation Undergoing Percutaneous Coronary Intervention; R, time to randomization; DAT, dual antithrombotic therapy; TAT, triple antithrombotic therapy; R15, rivaroxaban 15 mg od; P2Y12, P2Y12 receptor inhibitor; R2.5, rivaroxaban 2.5 mg bid; ASA, aspirin; VKA, vitamin K antagonist; D150, dabigatran 150 mg bid; D110, dabigatran 110 mg bid; A5, apixaban 5 mg bid; E60, edoxaban 60 mg od; TIMI, thrombolysis in myocardial infarction; BRMA, bleeding requiring medical attention; CRNMB, clinically relevant non-major bleeding; ISTH, International Society on Thrombosis and Haemostasis.

NOACs have been directly compared with VKA in NVAF patients undergoing PCI or with ACS. Figure 1 provides a summary of the rationale and design of the four representative RCTs, which include the PIONEER AF-PCI study (Open-Label, Randomized, Controlled, Multicenter Study Exploring Two Treatment Strategies of Rivaroxaban and a Dose-Adjusted Oral Vitamin K Antagonist Treatment Strategy in Subjects with Atrial Fibrillation who Undergo Percutaneous Coronary Intervention) (5), the RE-DUAL PCI study (Randomized Evaluation of Dual Antithrombotic Therapy with Dabigatran vs. Triple Therapy with Warfarin in Patients with non-valvular Atrial Fibrillation Undergoing Percutaneous Coronary Intervention) (6), the AUGUSTUS study (Open-label, Two-by-two Factorial, Randomized Controlled, Clinical Trial to Evaluate the Safety of Apixaban vs. Vitamin K Antagonist and Aspirin vs. Aspirin Placebo in Patients with Atrial Fibrillation and Acute Coronary Syndrome and/or Percutaneous Coronary Intervention) (7), and the ENTRUST-AF PCI study (Edoxaban Treatment vs. Vitamin K Antagonist in Patients with Atrial Fibrillation Undergoing Percutaneous Coronary Intervention) (8). Notably, all of these trials were under powered to evaluate outcomes of cardiac ischemic or cerebrovascular events.

### PIONEER AF-PCI Trial

The PIONEER AF-PCI study was the first RCT to analyze two different rivaroxaban strategies with VKA-treated standard triple strategy in 2,124 AF patients after PCI (Figure 1) (5). The study included three treatment arms: low-dose rivaroxaban [15 mg once daily (od), or 10 mg od in patients with creatinine clearance (CrCl) 30–50 ml/min] plus one P2Y12 inhibitor continuing for 12 months (group 1), a very low dosage of rivaroxaban [2.5 mg twice daily (bid)] plus DAPT for the next 1, 6, or 12 months, followed by rivaroxaban 15 mg od (or 10 mg od if renal impairment) plus aspirin for the rest time of the 12-month duration (group 2), or a conventional TAT with dose-adjusted VKA along with DAPT lasting for 1, 6, or 12 months, followed by VKA plus aspirin for the rest of 12-month duration (group 3) (13). The results of the study showed that patients allocated to both arms with rivaroxaban had a significantly reduced incidence of clinically significant bleeding at 12 months than did the VKA-based control group [group 1 vs. group 3: 16.8 vs. 26.7%; hazard ratio (HR) 0.59; 95% confidence interval (CI): 0.47–0.76; *p* < 0.001; group 2 vs. group 3: 18.0 vs. 26.7%; HR 0.63; 95% CI: 0.50–0.80; *p* < 0.001]. Moreover, no obvious differences in the incidence rates of cardiovascular death, myocardial infarction (MI), and stroke were observed in patients allocated to the different arms (group 1: 6.5%, group 2: 5.6%, and group 3: 6.0%; *p*-values for all comparisons were non-significant) (Table 1). In addition, the researchers stated that ~4,000 participants needed to be included to achieve adequate statistical power for efficacy endpoint evaluation, making it unlikely to be implemented (15). Additionally, the two doses of rivaroxaban (15 mg/10 mg od or 2.5 mg bid) used in the PIONEER AF-PCI trial have not yet been validated for stroke prevention in AF (SPAF). The J-ROCKET trial [Japan-specific rivaroxaban dose (15 mg od for normal renal function) in a Japanese population] was excluded, because the power of the study was not sufficient (16). It is worth mentioning that from the PIONEER AF-PCI trial, DAT was associated with a lower risk of re-hospitalization caused by bleeding or cardiovascular events than TAT (17). This finding lends further credence to the use of DAT for AF-ACS population.

**Table 1:** Clinical end point presentations with NOAC-based antithrombotic therapy regimens for AF patients undergoing PCI or with ACS: PIONEER AF-PCI, RE-DUAL PCI, AUGUSTUS, and ENTRUST-AF PCI trials.

**Safety end points**			**Event no. /total no. (%)**				
**PIONEER AF-PCI**			**TIMI bleeding category**				
			**Major bleeding**		**Minor bleeding**		**BRMA**
R15 + P2Y12			14/696 (2.0)		7/696 (1.0)		93/696 (13.4)
R2.5 + DAPT			12/706 (1.7)		7/706 (1.0)		102/706 (14.4)
VKA + DAPT			20/697 (2.9)		13/697 (1.9)		139/697 (19.9)
**RE-DUAL PCI**		**ISTH bleeding category**				**TIMI bleeding category**	
		**Major bleeding**		**CRNMB**	**ICH**	**Major bleeding**	**Minor bleeding**
D110 + P2Y12		49/981 (5.0)		102/981 (10.4)	3/981 (0.3)	14/981 (1.4)	15/981 (1.6)
VKA + DAPT		90/981 (9.2)		174/981 (17.7)	10/981 (1.0)	37/981 (3.8)	32/981 (3.2)
D150 + P2Y12		43/763 (5.6)		111/763 (14.6)	1/763 (0.1)	16/763 (2.1)	11/763 (1.4)
VKA + DAPT^a^	64/764 (8.4)		132/764 (17.3)		8/764 (1.0)	30/764 (3.9)	18/764 (2.4)
**AUGUSTUS**	**ISTH bleeding category**			**TIMI bleeding category**		**GUSTO category**	
	**Major bleeding**	**CRNMB**	**ICH**	**Major bleeding**	**Minor bleeding**	**Severe bleeding**	**Moderate bleeding**
A5 + P2Y12 ± ASA	69/2,290 (3.0)	180/2,290 (7.9)	5/2,290 (0.2)	38/2,290 (1.7)	80/2,290 (3.5)	5/2,290 (0.2)	37/2,290 (1.6)
VKA + P2Y12 ± ASA	104/2,259 (4.6)	246/2,259 (10.9)	13/2,259 (0.6)	48/2,259 (2.1)	118/2,259 (5.2)	8/2,259 (0.4)	64/2,259 (2.8)
A5/VKA + DAPT	108/2,277 (4.7)	275/2,277 (12.1)	8/2,277 (0.4)	55/2,277 (2.4)	126/2,277 (5.5)	7/2,277 (0.3)	63/2,277 (2.8)
A5/VKA + P2Y12	65/2,279 (2.9)	148/2,279 (6.5)	10/2,279 (0.4)	29/2,279 (1.3)	71/2,279 (3.1)	6/2,279 (0.3)	37/2,279 (1.6)
**ENTRUST-AF PCI**			**ISTH bleeding category**				
		**Major bleeding**			**CRNMB**		
	**ITT analysis**		**OT analysis**		**ITT analysis**		**OT analysis**
E60 + P2Y12	45/751 (6.0)		42/746 (5.6)		83/751 (11.1)		82/746 (11.0)
VKA + DAPT	48/755 (6.4)		42/740 (5.7)		104/755 (13.8)		100/740 (13.5)
**Efficacy end points**			**Event no./total no. (%)**				
**PIONEER AF-PCI**		**Composite efficacy end point**^**b**^		**CV Death**	**MI**	**Stroke**	**ST**
R15 + P2Y12		63/694 (9.1)		15/694 (2.2)	19/694 (2.7)	8/694 (1.2)	5/694 (0.7)
R2.5 + DAPT		64/704 (9.1)		14/704 (2.0)	17/704 (2.4)	10/704 (1.4)	6/704 (0.9)
VKA + DAPT		64/695 (9.2)		11/695 (1.6)	21/695 (3.0)	7/695 (1.0)	4/695 (0.6)
**RE-DUAL PCI**		**Composite efficacy end points**^**c**^		**Death**	**MI**	**Stroke**	**ST**
D110 + P2Y12		149/981 (15.2)		55/981 (5.6)	44/981 (4.5)	17/981 (1.7)	15/981 (1.5)
VKA + DAPT		131/981 (13.4)		48/981 (4.9)	29/981 (3.0)	13/981 (1.3)	8/981 (0.8)
D150 + P2Y12		90/763 (11.8)		30/763 (3.9)	26/763 (3.4)	9/763 (1.2)	7/763 (0.9)
VKA + DAPT		98/764 (12.8)		35/764 (4.6)	22/764 (2.9)	8/764 (1.0)	7/764 (0.9)
**AUGUSTUS**	**Hospitalization**	**Death**	**CV Death**	**Stroke**	**MI**	**ARC definite or probable ST**^**d**^	**Urgent revascularization**
A5 + P2Y12 ± ASA	518/2,306 (22.5)	77/2,306 (3.3)	57/2,306 (2.5)	13/2,306 (0.6)	72/2,306 (3.1)	14/2,306 (0.6)	40/2,306 (1.7)
VKA + P2Y12 ± ASA	607/2,308 (26.3)	74/2,307 (3.2)	54/2,308 (2.3)	26/2,308 (1.1)	80/2,308 (3.5)	18/2,308 (0.8)	44/2,308 (1.9)
A5/VKA + DAPT	585/2,307 (25.4)	72/2,307 (3.1)	53/2,307 (2.3)	20/2,307 (0.9)	68/2,307 (2.9)	11/2,307 (0.5)	37/2,307 (1.6)
A5/VKA + P2Y12	540/2,307 (23.4)	79/2,307 (3.4)	58/2,307 (2.5)	19/2,307 (0.8)	84/2,307 (3.6)	21/2,307 (0.9)	47/2,307 (2.0)
**Efficacy end points**							**Event no./total no. (%)**
**ENTRUST-AF PCI**							**Main efficacy outcomes^**e**^**
							**ITT analysis**
E60 + P2Y12							49/751 (6.5)
VKA + DAPT							46/755 (6.1)

*NOAC, non-vitamin K antagonist oral anticoagulant; AF, atrial fibrillation; PCI, percutaneous coronary intervention; ACS, Acute Coronary Syndromes; PIONEER AF-PCI, Open-Label, Randomized, Controlled, Multicenter Study Exploring Two Treatment Strategies of Rivaroxaban and a Dose-Adjusted Oral Vitamin K Antagonist Treatment Strategy in Subjects with Atrial Fibrillation who Undergo Percutaneous Coronary Intervention; RE-DUAL PCI, Randomized Evaluation of Dual Antithrombotic Therapy with Dabigatran vs. Triple Therapy with Warfarin in Patients with Non-valvular Atrial Fibrillation Undergoing Percutaneous Coronary Intervention; AUGUSTUS, Open-label, two-by-two Factorial, Randomized Controlled, Clinical Trial to Evaluate the Safety of Apixaban vs. Vitamin K Antagonist and Aspirin vs. Aspirin Placebo in patients with Atrial Fibrillation and Acute Coronary Syndrome and/or Percutaneous Coronary Intervention; ENTRUST-AF PCI, Edoxaban Treatment vs. Vitamin K Antagonist in Patients with Atrial Fibrillation Undergoing Percutaneous Coronary Intervention; R15, rivaroxaban 15 mg od; P2Y12, P2Y12 receptor inhibitor; R2.5, rivaroxaban 2.5 mg bid; ASA, aspirin; VKA, vitamin K antagonist; D110, dabigatran 110 mg bid; D150, dabigatran 150 mg bid; A5, apixaban 5 mg bid; E60, edoxaban 60 mg od; DAPT, dual antiplatelet therapy; TIMI, thrombolysis in myocardial infarction; BRMA, bleeding requiring medical attention; ISTH, International Society on Thrombosis and Haemostasis; ICH, intracranial hemorrhage; CRNMB, clinically relevant non-major bleeding; GUSTO, global use of strategies to open occluded arteries; ITT, intention-to-treat; OT, on-treatment; CV, cardiovascular; MI, myocardial infarction; ARC, Academic Research Consortium; ST, stent thrombosis*.

a *Corresponding matched triple treatment group with VKA*.

b *Composite efficacy endpoint of thromboembolic events (myocardial infarction, stroke, or systemic embolism), death, and unplanned revascularization from the PIONEER AF-PCI trial was reported in a separate substudy (14). This information has been added to allow for comparison with results from the RE-DUAL PCI trial*.

c *Composite efficacy end points were thromboembolic events, death, or unplanned revascularization*.

d *Excluding 1,097 patients with medically managed ACS*.

e *Main efficacy outcomes were composite of cardiovascular death, stroke, systemic embolic event, MI, or definite ST*.

### RE-DUAL PCI Trial

The RE-DUAL PCI study was the second full-scale RCT regarding the topic (Figure 1) (6), and it compared two dabigatran (110 mg or 150 mg bid)-based DAT regimens plus a P2Y12 inhibitor with a standard TAT containing VKA and DAPT in 2,725 AF patients undergoing PCI (18). As for the TAT duration, triple therapy was continued for 1 month for patients with a bare-metal stent and lasted 3 months for those with a drug-eluting stent (DES). During a mean follow-up duration of 14 months, this study showed that the incidence of the composite end point of major or clinical relevant non-major (CRNM) bleeding was significantly smaller in patients allocated to either of the dabigatran dual therapy arms than among those assigned to the triple therapy arm (dabigatran 110 mg regimen vs. triple therapy: 15.4 vs. 26.9%; HR, 0.52; 95% CI, 0.42–0.63, *p* < 0.0001 for non-inferiority, *p* = 0.0001 for superiority; dabigatran 150 mg regimen vs. corresponding triple therapy: 20.2 vs. 25.7%; HR, 0.72; 95% CI, 0.58–0.88; *p* < 0.0001 for noninferiority, *p* = 0.002 for superiority). Subsequently, data from patients of the two dabigatran arms were pooled to identify the composite effectiveness endpoints of death, thromboembolic events (MI, stroke, or systemic embolism) and unplanned revascularization. Dabigatran-based dual therapy was shown to be non-inferior to standard TAT (dabigatran dual therapy vs. warfarin triple therapy: 13.7 vs. 13.4%; HR, 1.04; 95% CI, 0.84–1.28) with respect to the efficacy endpoints, although the study was underpowered (Table 1). Furthermore, on the basis of the design and rationale of the PIONEER-AF PCI and the RE-DUAL PCI studies, we could not discriminate whether the decrease in bleeding should be ascribed to the use of NOAC vs. VKA, the avoidance of aspirin, or both factors (19).

### AUGUSTUS Trial

The AUGUSTUS trial was published in 2019 (7) and was designed as a two-by-two factorial RCT evaluating the safety and efficacy of omitting aspirin with both VKA and apixaban, against a background of combining P2Y12 inhibitor (most commonly clopidogrel) treatment for 6 months in 4,614 AF patients with a recent ACS event or undergoing PCI (Figure 1) (20). The AUGUSTUS study eagerly addressed crucial missing components of the RE-DUAL PCI, PIONEER AF-PCI and ENTRUST-AF PCI trials by performing comparisons within the triple antithrombotic regimens (apixaban plus P2Y12 inhibitor vs. VKA plus P2Y12 inhibitor) and the dual antithrombotic regimens (apixaban plus DAPT vs. VKA plus DAPT). Correspondingly, the outcomes of the study were expected to disentangle whether the efficiency of bleeding reduction was caused by the administration of the NOAC itself or aspirin avoidance. This is particularly important because both factors are constructive (21). The trial also included AF patients with medically-managed ACS who did not receive PCI, accordingly, which could therefore expand the present understanding for these patients (19). At 6 months, the primary endpoint of major or CRNM bleeding was observed in 10.5% of patients allocated to the apixaban group and 14.7% of patients in the VKA group (HR, 0.69; 95% CI, 0.58–0.81; *p* < 0.001 for non-inferiority and superiority), which was shown to be increased by aspirin as compared with placebo (aspirin vs. placebo: 16.1 vs. 9.0%; HR, 1.89; 95% CI, 1.59–2.24; *p* < 0.001). Even though the trial was underpowered to account for ischemic outcomes, the results showed that the apixaban-treated group experienced fewer cases of death or hospitalization than did the VKA-treated group (apixaban vs. VKA: 23.5 vs. 27.4%; HR, 0.83; 95% CI, 0.74–0.93; *p* = 0.002), while the incidences of death and ischemic events were not statistically different (apixaban vs. VKA: 6.7 vs. 7.1%; HR, 0.93; 95% CI, 0.75–1.16) (Table 1). In brief, the incidence of the primary outcome gradually decreased across the four treatment strategies in the following order: VKA plus DAPT (49.1 events per 100 patient-years) > apixaban plus DAPT (33.6 events per 100 patient-years) > VKA plus P2Y12 inhibitor (26.7 events per 100 patient-years) > apixaban plus P2Y12 inhibitor. This study clearly revealed that a DAT regimen containing apixaban plus a P2Y12 inhibitor (avoiding aspirin) was associated with reduced risks of bleeding and hospitalization, without a significant increase in ischemic events when compared to strategies such as a VKA, aspirin, or both. These findings highlight the importance of shareable, patient-oriented decision-making involving the appropriate course of antithrombotic regimen after ACS and/or PCI in patients with AF and with OAC, which has been strongly recommended by recent consensus documents (9, 11, 22).

### ENTRUST-AF PCI Trial

The ENTRUST-AF PCI study focused on the role of the edoxaban-based strategy in AF patients with ACS or those with stable coronary artery disease (CAD) undergoing PCI (Figure 1). Patients were randomly allocated to receive an edoxaban-based regimen (edoxaban 60/30 mg od combining a P2Y12 inhibitor) for 12 months or a combined TAT regimen with VKA, a P2Y12 inhibitor and aspirin (aspirin for 1–12 months) (23). With 1,506 patients included, the results of the study indicated that edoxaban-based DAT was non-inferior to VKA-based TAT regarding the risk of major or CRNM bleeding events [annualized event rate (edoxaban group vs. VKA group): 20.7 vs. 25.6%; HR 0.83, 95% CI 0.65–1.05, *p* = 0.0010 for non-inferiority, margin HR 1.20, *p* = 0.1154 for superiority]. Additionally, the incidence of the composite outcome of ischemic events [cardiovascular death, stroke, systemic embolic events (SEE), MI, and definite ST] also did not statistically differ between the groups (Table 1). Like other trials of NOAC-based regimens for AF with ACS/PCI, this study was also underpowered to evaluate differences in main efficacy endpoints (8). The bleeding events were numerically fewer among patients allocated to the edoxaban group, but no significant difference was detected between groups (Table 1). Subsequent Kaplan-Meier analysis indicated an unforeseen trend with an unfavorable HR for edoxaban vs. VKA within the first 2 weeks, while the HR consistently favored edoxaban over the remaining trial period. Importantly, the rates of bleeding events within the first 2 weeks of the study were comparable for patients allocated to the two treatments, suggesting the potential confounding effects of insufficient anticoagulation [the proportion of international normalized ratio (INR) <2: 69% for the first week, and 42% for the second week] in patients allocated to the TAT group (9).

## Recommendations from Guidelines and Consensus Documents for Antithrombotic Regimens in AF-PCI/ACS

In view of the accumulating evidence and experience pertaining to the optimal antithrombotic regimens in AF patients undergoing PCI or experiencing ACS, multiple guidelines and consensus documents have been produced in the last decades (24, 25). Originally, recommendations were mostly founded on expert consensus due to the lack of evidence-based RCTs. Subsequently, the evidence has been reinforced with the publication of a series of clinical trials, with some opinions discarded and others updated (19). As discussed previously, to illustrate the issues clearly, we consulted the 2019 AHA/ACC/HRS AF guideline for the management of AF (9) (for which the AUGUSTUS and ENTRUST-AF PCI trials were not referenced since the findings from these trials had not yet been released at the time), the 2020 ESC guidelines for the diagnosis and management of AF developed in collaboration with the EACTS (11), the 2020 ESC guidelines for the management of ACS in patients presenting without persistent ST-segment elevation (22), and the 2020 antithrombotic management of patients with atrial fibrillation and coronary artery disease: expert consensus document of Chinese Society of Cardiology (10). The following summaries also draw from guidelines and consensus documents updated from the North American, European and Chinese perspectives and are based on personalized solutions considering bleeding risk and ischemic risk. Moreover, current research hotspots and controversies regarding the optimal antithrombotic regimens in AF patients with PCI or ACS are also discussed.

### North American Perspective

“For AF patients at high risk of thrombosis with ACS, anticoagulation is endorsed unless bleeding risk exceeds the expected profits. If TAT (OAC, aspirin, and P2Y12 inhibitor) is prescribed for AF patients after PCI, it is rational to select clopidogrel rather than prasugrel. For AF patients with stenting for ACS, DAT (clopidogrel or ticagrelor plus VKA; clopidogrel plus rivaroxaban 15 mg od; clopidogrel plus dabigatran 150 mg bid) is expected to lower the bleeding risk compared with TAT. If TAT (OAC, aspirin, and a P2Y12 inhibitor) is used for AF patients with PCI for ACS, a switch to DAT (OAC and a P2Y12 inhibitor) at 4–6 weeks may be a consideration” (9).

### European Perspective

“If AF patients qualify for anticoagulation, we recommend NOAC-based regimen in preference to VKA plus antiplatelet therapy. During PCI, uninterrupted anticoagulant therapy with VKA or NOAC should be considered, and additional parenteral anticoagulation is recommended, without considering the time of the last administration of NOACs and whether the INR is <2.5 for VKA-treated patients. For patients with a high bleeding risk (HBR, HAS-BLED ≥3), rivaroxaban 15 mg od, or dabigatran 110 mg bid should be considered prior to rivaroxaban 20 mg od, or dabigatran 150 mg bid for the duration of concomitant single antiplatelet therapy (SAPT) or DAPT. When AF patients with VKA indication and receiving antiplatelet treatment, the VKA dose intensity should be cautiously adjusted within the proper INR range of 2.0–2.5 with a time in the therapeutic range (TTR) of >70%. For patients with AF complicated by ACS treated by uncomplicated PCI, early discontinuance of aspirin (up to 1 week from the acute incident) and DAT (NOAC plus a P2Y12 inhibitor, preferably clopidogrel) should be considered as one default strategy for up to 1 year if stent thrombosis (ST) risk is low or if bleeding risks exceed the ST risk, irrespective of the type of stent used, and OAC monotherapy should proceed thereafter. TAT [OAC, aspirin, and clopidogrel (not ticagrelor or prasugrel)] for more than 1 week and up to 1 month after ACS should be considered for those with high ischemic risk, or other anatomical/procedural characteristics that outweigh the bleeding risk, and strategies should be clearly stipulated at hospital discharge. For patients requiring an OAC, aspirin combined with clopidogrel for more than 1 week and up to 1 month should be considered in the patients with high ischemic risk or with other anatomical/procedural features that outweigh the risk of bleeding. DAT [OAC plus a powerful P2Y12 inhibitor (ticagrelor or prasugrel)] may be a consideration as one alternative to the TAT regimen (OAC, aspirin, and clopidogrel) in patients with medium or high ST risk, regardless of stent type. For uncomplicated PCI, early cessation of aspirin ( ≤ 1 week) and continued DAT (OAC plus a P2Y12 inhibitor) should be lasted for up to 6 months, and clopidogrel is recommended when ST risk is low, or when concerns about bleeding risk outweigh concerns of ST risk, irrespective of the type of stent used. For medically managed patients with AF, apixaban 5 mg bid plus SAPT (recommended clopidogrel) for at least 6 months may be a consideration” (11, 22).

### Chinese Perspective

“For the majority of patients with NVAF after PCI, NOAC should be preferred to VKA If there are no contraindications. NOAC should be administered based on the verified dosing recommendations from published RCTs (PIONEER AF-PCI, RE-DUAL PCI, AUGUSTUS and ENTRUST-AF PCI trials). The antithrombotic program should be founded on an individualized approach accounting for ischemia and bleeding risk. When considering applying DAT regimen, the approach has been to continue aspirin (TAT strategy) until discharge. For patients with higher ischemia or thromboembolism risk and accompanying with low bleeding risk, aspirin can continue to keep until 1 month after PCI, but rarely more than 1 month. Since both ticagrelor and prasugrel have been correlated with a greater bleeding risk than that with clopidogrel, preference should be considered to clopidogrel in such patients requiring PCI, although some support for ticagrelor in this circumstance as well. Most patients with DAT regimen should consider discontinuing antiplatelet therapy at 1 year after PCI. For patients deemed to be at high bleeding risk and with low ischemic risk, discontinuation of antiplatelet therapy needs to be considered after 6 months for those accepting PCI; and for patients with low bleeding risk but high ischemic risk, it may be reasonable to continue DAT regimen after 1 year of PCI. The expert panel suggests starting or continuing a proton pump inhibitor (PPI, such as pantoprazole or rabeprazole) along with avoidance of concomitant non-steroidal anti-inflammatory drugs (NSAIDs) to reduce the risk of gastrointestinal (GI) bleeding after coronary stent implantation.” (10).

**Figure 2:**
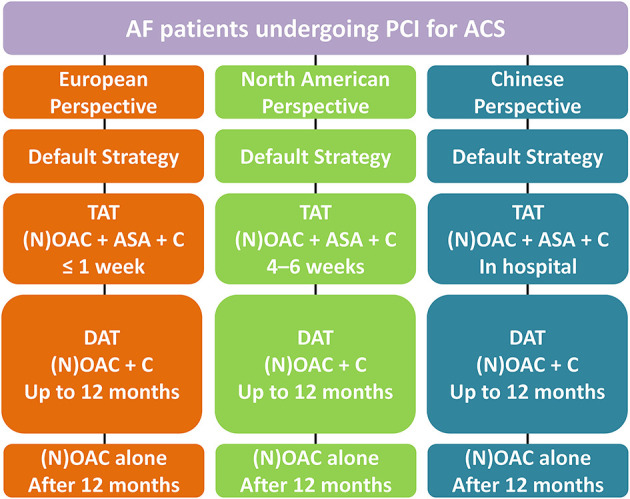
Antithrombotic management in AF patients undergoing PCI for ACS. AF, Atrial fibrillation; PCI, Percutaneous coronary intervention; ACS, Acute coronary syndrome; TAT, triple antithrombotic therapy; NOAC, non-vitamin K antagonist oral anticoagulant; OAC, Oral anticoagulation; ASA, aspirin; C, clopidogrel; DAT, dual antithrombotic therapy.

Hence, the European perspective so far is to recommend an early discontinuance of aspirin (generally ≤ 1 week) and continuation of the DAT regimen with NOAC plus clopidogrel. This differs from the North American viewpoint, which suggests TAT strategy for 4–6 weeks as a feasible approach based on net-benefit for reducing the ischemia and bleeding risks, whereas Chinese proposal recommends stopping aspirin (TAT strategy) at the day of discharge (Figure 2).

## Critical Discussions of Key Scientific Data

Published reports from the PIONEER AF-PCI, RE-DUAL PCI, AUGUSTUS and ENTRUST-AF PCI trials have challenged the position of conventional VKA-based TAT regimen as a standard care for AF patients receiving PCI and/or with ACS. When choosing the optimal antithrombotic regimens for such patients, physicians should carefully consider the risks and benefits of such therapies (26). Currently, the general strategy is to keep OAC and to adjust the intensity and the duration of antiplatelet treatment. In addition, antiplatelet therapy should be avoided in AF patients without definite indications, such as those with CAD or beyond 12 months after an acute coronary event.

In terms of OAC category, NOACs as part of antithrombotic combinations may be preferred to VKAs because of the lower bleeding risk. Moreover, the DAT regimen (without aspirin) has been confirmed to be linked to a remarkable reduction in major bleeding as compared to TAT, although the benefits for ischemic events were similar. A network meta-analysis including the above four trials confirmed that the potential reduction in major bleeding was even greater compared to TAT (with VKA if NOACs were incorporated into the DAT regimen in these patients; relative risk reduction: 37 vs. 31%) (4). The homogeneity of the results from the NOAC trials highlighted their benefits in reducing bleeding risk in patients with AF undergoing PCI. In fact, this observation could be explained by the fact that continuing VKA is practically more of a problem, since the target of INR range must be carefully modulated (target INR: 2.0–2.5 and TTR > 70%) during treatment with a VKA plus antiplatelet therapy combination (11, 22), which may lead to a relatively higher risk of bleeding in patients with TAT including VKA. Consistently, the 2019 ESC guidelines recommended that for AF after PCI or with other indications for OAC, a NOAC is preferable to VKA when combined with antiplatelet agents (IA) (27).

The trial-determined secondary outcomes [ischemic end points, which included major adverse cardiovascular events (MACE), composite of death, thromboembolic events, etc.] were similar among all treatment arms (28). However, because the four trials were underpowered to evaluate the risk of thrombosis, it remains unclear whether DAT (i.e., rivaroxaban 15 mg od/2.5 mg bid, dabigatran 110/150 mg bid, apixaban 5 mg bid, or edoxaban 60 mg od plus a P2Y12 inhibitor) is adequate for the prevention of ST or MI in patients undergoing PCI. In the RE-DUAL PCI trial, numerically, the incidence of MI or ST was slightly higher for patients allocated to receive dabigatran 110 mg bid than for those given dabigatran 150 mg bid (6). Although the difference was not significant, a dosage of 150 mg bid may be preferable for high-ischemic patients with PCI if dabigatran is chosen (29). Additionally, regarding the prevention for ST, some controversy remains in patients regarding DAT (NOAC plus a P2Y12 inhibitor and with no aspirin) vs. TAT within the first week after stent placement (12, 30). Interestingly, another meta-analysis showed that the risk of ST is not statistically higher in patients receiving DAT compared to that in patients receiving TAT (VKA + DAPT) (4). This may be explained by the fact that approximately one-third of the participants in the AUGUSTUS trail never underwent stent implantation at the index event. Accordingly, there was no ST risk for these patients (31). Another study incorporated data regarding ST incidence in the AUGUSTUS trial of only patients receiving stents, which might cause divergent results (4).

The optimal TAT duration for AF patients after PCI or with ACS remains disputed. Nevertheless, regardless of whether a DAT or TAT strategy is applied, the ultimate intention is to optimally balance the preventative efficacy of thrombosis and avoidance of antithrombotic-related bleeding complications (2). The WOEST (What is the Optimal antiplatElet and anticoagulant therapy in patients with oral anticoagulation and coronary StenTing) study was the first pivotal RCT to estimate the efficacy of aspirin omission after PCI on safety endpoints as compared with standard TAT (aspirin, clopidogrel, and VKA). It showed that DAT (clopidogrel and VKA) was associated with significantly decreased bleeding complications (32). In the ISAR-TRIPLE (Triple Therapy in Patients on Oral Anticoagulation After Drug Eluting Stent Implantation) trial, patients treated with drug-eluting stent implantation and then OAC and aspirin were randomly assigned to accept a 6-week or 6-month course of clopidogrel. The results showed no significant difference in a composite outcome of death, MI, definite ST, stroke, or TIMI major bleeding at 9 months. These results suggest that short-term treatment with TAT may be safe in ACS/PCI patients requiring an OAC (33). However, both the WOEST and ISAR-TRIPLE studies were small and underpowered to evaluate the thromboembolic outcomes. Although these two trials have limitations, simplification of a VKA-based TAT strategy by aspirin withdrawal or shortening DAPT duration may be considered in future studies.

Subsequently, the findings from the four NOAC-based trials have challenged the role of TAT (VKA as standard procedure) in AF patients who have had a recent ACS event or are undergoing PCI. In contrast to the early hypothesis, long-term TAT does not seem to be mandatory for these patients. Interestingly, we have noted that the mean time from the index event to enrollment varied among the four RCTs (5–8): ≤ 3 days in the PIONEER AF-PCI study, ≤ 5 days in the RE-DUAL PCI and ENTRUST-AF PCI studies, and ≤ 14 days in the AUGUSTUS study (hence a brief duration of TAT may be appropriate) (4). Given the potential ST risk prevention with DAT, it can be hypothesized that patients at an immediate or early stage of ACS or PCI are more likely to benefit from the initial short span of TAT (34). Because details on the timing of ST were not entirely available among the four trials, it may be too early to draw a conclusion regarding the optimal duration for aspirin plus NOAC and with a P2Y12 (35). According to the available evidence, the duration of TAT should generally be minimized to the extent possible (preferred ≤ 1 week), especially in patients with an increased risk of bleeding. Conversely, for patients with a high ischemia risk and a low bleeding risk, it may be desirable to continue aspirin for 1 month, because this is the time of greatest ST risk. This is particularly of significance for patients with ACS or recurrent MI, complex revascularization, multi-vessel stents, chronic kidney disease [CKD, estimated glomerular filtration rate (eGFR) <60 ml/min], etc. (22).

Another critical issue that is worth addressing is the selection of the P2Y12 inhibitor. Clopidogrel remains the most frequently used P2Y12 inhibitor (overall > 90%) in combination with NOAC, while data for the use of prasugrel or ticagrelor in combination of NOAC remain limited (5–8). In the RE-DUAL PCI trial (6), 12% of patients were given ticagrelor, and thus, the results provide some insights regarding therapy efficiency. However, the corresponding percentages were only 4.3% in the PIONEER AF-PCI trial, 6.2% in the AUGUSTUS trial, and 7.0% in the ENTRUST-AF PCI trial. For patients with a low bleeding risk and complicated thrombotic factors, ticagrelor plus a NOAC may be a potential choice. The experience of prasugrel use was also limited (1.3% in the PIONEER AF-PCI study, 1.1% in the AUGUSTUS study, <1% in the ENTRUST-AF PCI study, and 0% in the RE-DUAL PCI trial) (5–8). Particularly, data for ticagrelor or prasugrel as DAT or TAT seem discouraging due to the high bleeding rate compared with that observed with clopidogrel (ticagrelor: relative risk [RR] 1.36; 95% CI, 1.18–1.57 and prasugrel: RR 2.11; 95% CI, 1.34–3.30) (36), which suggests that powerful P2Y12 inhibitors may not be the first-line choice for use with OAC in AF patients after PCI. Because clopidogrel is characterized as providing less potent and variable platelet inhibition (37), more individualized and comprehensive antithrombotic strategies could be evaluated in future studies.

Moreover, it remains unclear whether the efficacy for SPAF is similar between lower doses of rivaroxaban (i.e., 2.5 mg bid, or 15 mg od) and antiplatelet agents, at least by comparison to dose-adjusted VKA or compared to a full dose of rivaroxaban (20 mg od) in patients with normal renal clearance (CrCl > 50 mL/min) (28). In the PIONEER AF-PCI study (5), there was no evidence that stroke risk was increased in the rivaroxaban group compared with the VKA-based group (15 mg rivaroxaban regimen 1.3 vs. 1.2% with VKA regimen, HR 1.07, 95% CI 0.39–2.96; 2.5 mg rivaroxaban regimen 1.5 vs. 1.2% with VKA regimen, HR 1.36, 95% CI 0.52–3.58). Moreover, it is still unknown whether DAT (i.e., rivaroxaban 15 mg od, or dabigatran 110/150 mg bid, apixaban 5 mg bid, or edoxaban 60 mg od plus a P2Y12 inhibitor) can adequately prevent ST or MI, because these trials were underpowered for comparisons between individual medications (27). When evaluated alone, efficacy endpoints were approximately 10-fold less frequent in current clinical practice compared to the safety results (bleeding outcomes), which were treated as primary endpoints in these studies (30).

For patients with chronic coronary syndrome (CCS) (>1 year ACS or PCI, without acute events) who were optimally managed medically, the available evidence supports continuation with a single OAC at full SPAF doses thereafter (9, 11, 22, 28). For instance, if a rivaroxaban-based low-dose regimen (e.g., 15 mg od/ 10 mg od with renal impairment) was selected, the full dose anticoagulant regimen (20 mg od/15 mg od with renal insufficiency) should be resumed after the withdrawal of antiplatelet treatment. Although the exact underlying mechanisms remain unknown, from the pathophysiological perspective, it may be rational since thrombin is the most powerful stimulus of platelet activation/aggregation (38, 39). In the COMPASS (Cardiovascular OutcoMes for People using Anticoagulation StrategieS) study (40, 41), by direct inhibition of factor Xa, patients receiving low-dose rivaroxaban (2.5 mg bid) plus aspirin showed better cardiovascular outcomes than those who were treated with aspirin alone. The AFIRE (Atrial Fibrillation and Ischemic Events with Rivaroxaban in Patients with Stable Coronary Artery Disease) trial also evaluated the role of NOAC monotherapy in AF patients with stable CAD (42, 43). In this study, the efficacy and safety of rivaroxaban alone were compared with those of a combined therapy with rivaroxaban plus a single antiplatelet agent in 2,236 AF patients after PCI or coronary-artery bypass grafting (CABG) for more than 1 year. The study was discontinued early because of higher mortality in the dual-therapy group. The results showed that rivaroxaban monotherapy (15 mg od with normal renal function or 10 mg od with CrCl 15–49 mL/min) was non-inferior to dual-therapy to prevent a composite efficacy outcome of stroke, systemic embolism, MI, unstable angina (UA) requiring revascularization, and total death (HR 0.72, 95% CI 0.55–0.95; *p* < 0.001 for non-inferiority). Furthermore, rivaroxaban monotherapy was associated with less major bleeding (HR 0.59, 95% CI 0.39–0.89; *p* = 0.01 for superiority) than dual-therapy. Thus, OAC monotherapy may be sufficient for AF patients with stable CAD (comprising chronic stage of PCI), adding an antiplatelet agent might increase the bleeding risk and doesn't provide additional benefit for thrombosis event prevention.

## Outlook

Further studies of antithrombotic therapy in AF patients undergoing PCI or with ACS are urgently warranted to provide more data, particularly for the issues described below. First, no RCTs have been performed to evaluate the efficacy of a predictive model of bleeding or ischemic risk in these patients (34). Second, the optimal timing of aspirin cessation has not been determined in patients receiving TAT. Therefore, for physicians, decisions regarding the appropriate antithrombotic agents and therapeutic duration should be made based on cautious evaluation of the individual patient's risks of ischemic and bleeding events, procedure considerations, patient compliance, as well as recommendations of evidence-based guidelines. Next, the optimal selection of antiplatelet drugs, either a potent P2Y12 inhibitor (e.g., ticagrelor, prasugrel) or aspirin, has not been sufficiently verified for different antithrombotic combinations. Fourth, the optimal antithrombotic regimens in patients with PCI requiring OAC for other indications, such as such as mechanical valve, venous thromboembolism (VTE), or left atrial thrombosis, remain to be determined. Additionally, the efficacy of DAT or TAT in “high-risk” patients, such as AF patients with complex re-vascularization, recurrent coronary ischemic events, or MI, should also be evaluated. In addition, different types of NOACs presented various benefit-to-risk potential. The potential of “tailored” solutions on the basis of individual characteristics in this era should be investigated in the future (35). Finally, none of the four trials were of adequate statistical power to evaluate the ischemic endpoint, and it remains unknown whether NOAC-based DAT can sufficiently prevent MI or ST (1). Considering the huge sample size required to detect a potential difference in efficacy outcome, a patient-level meta-analysis based on the available evidence would be of significance (19).

## Conclusions

Post-procedural antithrombotic management in AF patients undergoing PCI or with ACS has been turning to a “less may be more” concept framework. The current paradigm favors NOAC in combination with a P2Y12 inhibitor (especially clopidogrel), which shows a better risk-benefit ratio than conventional VKA-based TAT regimens. Regarding aspirin, it could be administered during early periprocedural period to decrease the risk of early-onset ischemic events. However, the duration should be as short as possible to reduce the bleeding risk.

## Data Availability Statement

The raw data supporting the conclusions of this article will be made available by the authors, without undue reservation.

## Author Contributions

SZ and PM proposed the concept and designed the research. SZ wrote the manuscript. XH, HC, ML, and BL modified the manuscript for critical details. All authors contributed to the article and approved the submitted version.

## Conflict of Interest

The authors declare that the research was conducted in the absence of any commercial or financial relationships that could be construed as a potential conflict of interest.
